# Effect of lurbinectedin on the QTc interval in patients with advanced solid tumors: an exposure–response analysis

**DOI:** 10.1007/s00280-020-04153-6

**Published:** 2020-10-27

**Authors:** Salvador Fudio, Josep Tabernero, Vivek Subbiah, Sant P. Chawla, Victor Moreno, Federico Longo, Rafael Lopez, Antonio Anton, Jose Manuel Trigo, Geoffrey Shapiro, Woondong Jeong, Victor Manuel Villalobos, Rubin Lubomirov, Carlos Fernandez-Teruel, Vicente Alfaro, Valentina Boni

**Affiliations:** 1 PharmaMar, Avda. De los Reyes, 1, Pol. Ind. La Mina-Norte, Colmenar Viejo, Madrid, 28770 Spain; 2 Vall d’Hebrón University Hospital and Institute of Oncology (VHIO), 08035 Barcelona, Spain; 3 The University of Texas MD Anderson Cancer Center, Houston, TX 77030 USA; 4 Sarcoma Oncology Center, Santa Monica, CA 90403 USA; 5 Fundación Jiménez Díaz, 28040 Madrid, Spain; 6 Hospital Ramon y Cajal, 28034 Madrid, Spain; 7 IDIS, CIBERONC, Hospital Clínico Universitario de Santiago de Compostela, 15706 Santiago De Compostela, Spain; 8 Hospital Universitario Miguel Servet, 50009 Zaragoza, Spain; 9 Hospital Virgen de la Victoria, 29010 Málaga, Spain; 10 Dana-Farber Cancer Institute, Boston, MA 02215 USA; 11 Cancer Therapy and Research Center, San Antonio, TX 78229 USA; 12 University of Colorado, Denver, CO 80045 USA; 13 START Madrid-CIOCC, Hospital Universitario San Chinarro, 28050 Madrid, Spain

**Keywords:** Lurbinectedin, QTcF, ECG, Plasma concentration, Cardiac repolarization, Effect compartment

## Abstract

**Purpose:**

This study assessed the effect of lurbinectedin, a highly selective inhibitor of oncogenic transcription, on the change from baseline in Fridericia’s corrected QT interval (∆QTcF) and electrocardiography (ECG) morphological patterns, and lurbinectedin concentration–∆QTcF (C-∆QTcF) relationship, in patients with advanced solid tumors.

**Methods:**

Patients with QTcF ≤ 500 ms, QRS < 110 ms, PR < 200 ms, and normal cardiac conduction and function received lurbinectedin 3.2 mg/m^2^ as a 1-h intravenous infusion every 3 weeks. ECGs were collected in triplicate via 12-lead digital recorder in treatment cycle 1 and 2 and analyzed centrally. ECG collection time-matched blood samples were drawn to measure lurbinectedin plasma concentration. No effect on QTc interval was concluded if the upper bound (UB) of the least square (LS) mean two-sided 90% confidence intervals (CI) for ΔQTcF at each time point was < 20 ms. C-∆QTcF was explored using linear mixed-effects analysis.

**Results:**

A total of 1707 ECGs were collected from 39 patients (females, 22; median age, 56 years). The largest UB of the 90% CI of ΔQTcF was 9.6 ms, thus lower than the more conservative 10 ms threshold established at the ICH E14 guideline for QT studies in healthy volunteers. C-∆QTcF was better fit by an effect compartment model, and the 90% CI of predicted ΔQTcF at C_max_ was 7.81 ms, also below the 10 ms threshold of clinical concern.

**Conclusions:**

ECG parameters and C-ΔQTcF modelling in this prospective study indicate that lurbinectedin was not associated with a clinically relevant effect on cardiac repolarization.

## Introduction

Lurbinectedin (Zepzelca™), also known as PM01183, is a highly selective inhibitor of oncogenic transcription, with in vitro activity in the low nanomolar range [1]. Lurbinectedin was approved in June 2020 by the U.S. Food and Drug Administration (FDA) to treat adult patients with metastatic small cell lung cancer with disease progression on or after platinum-based chemotherapy [2]. Lurbinectedin inhibits the transcription process through (i) its binding to CG-rich sequences, mainly located around promoters of protein-coding genes; (ii) the irreversible stalling of elongating RNA polymerase II on the DNA template and its specific degradation by the ubiquitin/proteasome machinery; and (iii) the generation of DNA breaks and subsequent apoptosis [3].

Lurbinectedin is highly protein-bound. Based on in vitro studies, metabolism by cytochrome P450 (CYP) 3A is the major clearance mechanism (*data on file*).

A population pharmacokinetic (PopPK) model [4] was developed with data from 443 cancer patients treated in six phase I and three phase II trials with 1-h intravenous (i.v.) infusion of lurbinectedin as a single agent or combined with other agents. The population estimate for total plasma clearance was 11.2 L/h, corresponding to a blood CL of ~ 17 L/h, thus reflecting a low extraction ratio of 0.19. The population estimate of apparent volume at steady state was 438 L. Inter-individual variability was moderate for all parameters, ranging from 20.9 to 51.2%. High α-1-acid glycoprotein and C-reactive protein, and low albumin reduced clearance by 28%, 20%, and 20%, respectively. Co-administration of cytochrome CYP3A inhibitors reduced clearance by 30%.

Predictable and reversible myelosuppression, particularly neutropenia, is the most common limiting toxicity for lurbinectedin [5].

The non-clinical cardiovascular safety pharmacology evaluation of lurbinectedin consisted of in vitro and in vivo studies (*data on file*). The half maximal inhibitory concentration (IC_50_) determined for lurbinectedin in an in vitro hERG assay was 8.8 μM (6.9 μg/mL), far above from the maximum plasma concentration (C_max_) reached in patients at therapeutic exposure (106 μg/mL). The in vivo studies were conducted in telemetered dogs and cynomolgus monkeys receiving a single i.v. bolus injection at the maximum tolerated dose. No effects were observed on lead II electrocardiogram (ECG) variables [PR, QT, and QTcF (Fridericia’s corrected QT)] and QTcV (QTc according to Van de Water’s formula) intervals, and QRS duration, ECG gross morphology, or cardiac rhythm. Lurbinectedin-related cardiovascular changes were limited to mild decreases in blood pressure and increased heart rate (HR) associated with drug-induced nausea, vomiting, and/or pain.

To date, no cardiac toxicity concerns (i.e., contractibility, conduction/rhythm, or repolarization alterations) have been identified with lurbinectedin as a single agent.

Lurbinectedin is an antitumor drug that cannot be administered to healthy subjects. Therefore, QT evaluation had to be performed in a cancer patient population at a therapeutic dose. This QT evaluation study was nested into a basket clinical trial that was conducted to determine whether lurbinectedin had any effects on the QT interval or any other ECG parameter, in patients with solid tumors at the recommended dose of 3.2 mg/m^2^ administered q3wk as a 1-h i.v. infusion, and not receiving any concomitant medication known to prolong QT interval. This population, with advanced solid cancer and several co-morbidities, allowed assessment of the QTc interval in a real-life population similar to the population for which therapeutic use of lurbinectedin is now approved.

## Materials and methods

Patients were recruited at 12 investigational sites in the U.S. and Spain. The study protocol was submitted to the QT Interdisciplinary Review Team at the U.S. FDA, which considered the tested dose reasonable, and the ECG/pharmacokinetic (PK) collection, sample size, and study design acceptable to fulfil the aims of the study. The study protocol was approved by the Independent Local Ethics Committee of each participating center and was conducted in accordance with the Declaration of Helsinki, Good Clinical Practice guidelines, and local regulations on clinical trials. Signed informed consent was obtained from all patients prior to any study-specific procedure.

### Eligibility criteria

Eligibility criteria included: patients ≤ 65 years old; Eastern Cooperative Oncology Group performance status (ECOG PS) ≤ 1; 12-lead ECG recorded between day -10 and day -2 before first lurbinectedin administration, consistent with normal cardiac conduction and function, that was read by a central laboratory, showing sinus rhythm, heart rate (HR) between 45 and 100 beats per min (bpm), QTcF ≤ 500 ms (ms), QRS interval < 110 ms, and PR interval < 220 ms; systolic blood pressure 90-150 mmHg and diastolic blood pressure ≤ 90 mmHg; and grade ≤ 1 serum electrolyte levels according to the National Cancer Institute Common Terminology Criteria for Adverse Events (NCI-CTCAE) v.4.0.

Patients were excluded if they had heart rhythm disturbances (e.g., atrial fibrillation), unusual T wave and U wave morphology, personal or family history of long QT syndrome, ECG findings of complete left bundle branch block, permanent ventricular pacemaker, or Brugada syndrome; significant ischemic coronary disease, New York Heart Association class III or IV congestive heart failure, myocardial infarction, or unstable angina within the last 6 months; any skin condition likely to interfere with ECG electrode placement, or history of breast implant or thoracic surgery likely to cause abnormality in electrical conduction; or prior exposure to anthracyclines at a cumulative dose of doxorubicin (or equivalent) > 450 mg/m^2^. Patients were also excluded if they were receiving QT-prolonging medication that could not be interrupted at least 48 h before each ECG assessment.

### Study design

This was a QT evaluation study (EudraCT No. 2015-000206-18; *ClinicalTrials.gov: NCT02451007*) performed at a subset of sites participating in a multicenter, open-label, exploratory, phase II basket clinical trial (EudraCT No: 2014-003773-42; *ClinicaTrials.gov: NCT02454972*) conducted in patients with selected advanced solid tumors. The schedule used in this QT evaluation study was that evaluated in the basket trial: lurbinectedin 3.2 mg/m^2^ given as a 1-h i.v. infusion q3wk.

Patients were instructed to avoid beverages containing alcohol or methylxanthine-containing products (e.g., chocolate bars/candies and beverages like hot chocolate, coffee, tea, or colas) for 24 h before each ECG assessment; consume standard meals while at the study site, but avoid spicy foods and excessive food consumption.

### Electrocardiogram acquisition and analysis

A screening ECG was collected between day -10 and day -2 before the first lurbinectedin infusion (day 1 of cycle 1) and transmitted to the central ECG laboratory to confirm the patient’s eligibility.

On day 1 of cycle 1, two baseline triplicate ECGs (three 10-s digital ECGs in close succession) were collected: one before administration of prophylactic medication or pre-dose 1, and the other after antiemetic prophylactic medication (palonosetron 0.25 mg i.v.) and before the start of the lurbinectedin infusion or pre-dose 2 (also on cycle 2).

In cycle 1 and cycle 2, the following triplicate ECGs were collected 5–10 min before their time-matched PK time points: 5 min before end of the lurbinectedin infusion (EOI), 30 min, 1, 3, 24, 72 (only on cycle 1), and 168 h after EOI.

A 12-lead automated digital ECG recorder [Mortara Instrument (Milwaukee, WI, USA) ELI-150 ECG 12-lead digital recorder] was provided by a third-party central ECG laboratory (eResearchTechnology, Inc., Philadelphia, PA, USA). Analysis and reporting of ECG data were performed by a limited number of skilled readers who were blinded to treatment time point. ECG review of a particular patient was performed by a single reader. Interval duration measurements were collected using computer-assisted caliper placements on three consecutive beats. A cardiologist then verified the interval durations and performed the morphology analysis, noting any T-U wave complex compatible with an effect on cardiac repolarization. The ECG analysis was conducted in Lead II or in Lead V5 if Lead II was not analyzable. If Lead V5 was not analyzable, then Lead V2 was used, and followed by the most appropriate lead if necessary. The mean of triplicate ECG measures at each time point for each patient was used for analyses.

### QT correction methods

Fridericia’s formula [6] is currently considered the most accurate method for correcting the effect of HR on QT interval [7], and was used as the main method for HR correction for QT (QTcF). Nevertheless, graphical inspection of QTcF and QT corrected by Bazett’s formula (QTcB) [8] versus RR interval plots and statistical comparison of resulting squared linear regression slopes (*R*^2^) was performed.

### Pharmacokinetic assessments

Pharmacokinetic (PK) profiles of lurbinectedin were obtained on cycle 1 and cycle 2. Following ECGs’ acquisition, blood samples were collected into K3EDTA tubes. Tubes were gently inverted several times and centrifuged at 2000 × g for 10 min at 4 °C to separate the plasma.

Plasma concentrations of lurbinectedin were measured by a validated liquid chromatography–mass spectrometry assay (*Dynakin S.L., Spain*), with ranges of 0.1–50 µg/L. The within- and between-day precisions ranged from 2.7 to 12.9% and from 5.1 to 10.7%, respectively. The within- and between-day accuracy (bias) ranged from 10 to 12% and from 5 to 6%, respectively.

Datasets were prepared using SAS Enterprise Guide v.7.11 HF3 (SAS Institute Inc., Cary, NC, USA). Non-compartmental PK parameters were determined using Phoenix WinNonlin v.6.3 (Certara, USA). Non-linear mixed-effect modelling was performed in NONMEM v.7.3.0 (GloboMax LLC, Hanover, MD, USA). Graphical and all other statistical analyses were performed in R v.3.2.5 (R Foundation for Statistical Computing, Vienna, Austria).

### Safety assessment

Cardiac adverse events (AEs) observed in the period of this QT evaluation study were evaluated and graded according to the National Cancer Institute Common Terminology Criteria for Adverse Events (NCI-CTCAE), v.4 and coded using the Medical Dictionary for Regulatory Activities (MedDRA) v.16.0. Laboratory results of particular relevance (i.e., changes in albumin, calcium, potassium, or magnesium) were graded according to the NCI-CTCAE v.4.

### Endpoints

The primary endpoint was the change in QTc corrected by the Fridericia’s formula (ΔQTcF) between each scheduled post-baseline ECG time point and baseline at cycle 1. A patient was considered evaluable for the primary endpoint if he/she had baseline and one or more post-baseline ECG assessments.

Secondary endpoints were the ΔQTcF/lurbinectedin plasma concentration relationship, and change in other ECG parameters (i.e., HR, QRS, and PR).

### Statistical methods

#### “By time point” analysis

The primary comparison was ΔQTcF at each ECG time point. A non-inferiority criterion of 20 ms was used to establish the absence of post-baseline QTc prolongation when compared to baseline. An analysis of variance model with mixed effects was fitted, with ΔQTc data as the dependent variable and ECG time point as the fixed effects, and patient as random effect.

Using the estimated least square means (LSM) and intra-patient standard deviation (Std) obtained from this model, a two-sided 90% confidence interval (CI) was calculated for each LSM ΔQTc. Non-inferiority was to be concluded if the upper bound (UB) of the one-sided 95% CI fell below 20 ms at each ECG time point.

#### Sample size calculation

Assuming that the intra-subject Std for change from baseline in QTc (ΔQTc) is 30 ms and that the true difference between means is 5 ms, a sample size of at least 25 evaluable patients in more than 80% of the scheduled post-baseline ECG time points *t* was planned to have 80% power to show that the UB of the two-sided 90% CI (UB of the one-sided 95% CI) for mean ΔQTc at each ECG time point was < 20 ms. Approximately 35 patients were to be enrolled to ensure that at least 25 evaluable patients completed all required assessments.

#### Concentration–QTc analysis

C–ΔQTcF was assessed using a linear mixed-effects (LME) model, as proposed by Garnett et al. [9]. As the data available came from a single arm study, the LME model was characterized by the intercept ($\theta_{1}$) and the slope ($\theta_{2}$), and their corresponding variabilities.

1 $$\Delta QTcF_{ij} = \left( {\theta_{1} \cdot e^{{\eta_{1,i} }} } \right) + \left( {\theta_{2} \cdot e^{{\eta_{2,i} }} } \right) \cdot C_{ij} + \theta_{3} \cdot Cycle + {\epsilon_{ij}} ,$$

where *C*_*ij*_ is the model-predicted lurbinectedin total plasma concentrations (or the lurbinectedin concentrations in the effect compartment if a hysteresis is present), and $\eta_{1,i}$ and $\eta_{2,i}$ are the random effects associated with the intercept term $\theta_{1}$ and the slope term $\theta_{2}$, respectively, and are assumed to be exponential, independent, and normally distributed. Furthermore, ${\epsilon_{ij}}$ is the random residual variability, assumed to be an additive, independent, and normally distributed random variable. In the absence of placebo data, the unstructured placebo model consisting on the fixed effect parameters accounting for treatment-specific intercept (*TRT*), mean ΔQTcF at each time point evaluated (*TIME*), and baseline QTc was not included in the model. Based on the fact that ECG and PK assessments in this study were performed on two occasions (cycle 1 and cycle 2), the effect of cycle $\theta_{3}$ was also assessed in the models. To select the model for the C-ΔQTcF analysis (i.e., direct or indirect), a potential delay of the ΔQTcF effect relative to plasma concentrations (hysteresis) was graphically and statistically assessed, as proposed by Darpo et al. [10]. To enable an adequate comparison of the model fit between the direct effect model and the effect compartment model, the model-based predicted total plasma concentrations at central compartment (“direct” model) and at the effect compartment (“indirect” model) were used. These were based on the individual PK parameters, obtained through a maximum a posteriori estimation based on the individual total plasma concentration available for each subject and a population PK model previously developed [4].

The assumption of linearity of the C–ΔQTcF relationship was assessed by goodness-of-fit plots. Besides, a model with an empirical quadratic term of total plasma concentration was fitted and the significance of the quadratic term was tested. In case of the absence of trends in the goodness-of-fit plots and a non-significant quadratic term, the C–∆QTcF relationship was considered linear; otherwise, the relationship was considered non-linear.

The predicted effect of lurbinectedin on ΔQTcF was estimated at the C_max_ geometric mean either at the central or at the effect compartment in the first two cycles following the administration of 3.2 mg/m^2^ i.v. over 1-h q3wk and was calculated as the estimated intercept ($\theta_{1,Est}$) plus the product of the estimated slope ($\theta_{2,Est}$) and geometric mean C_max_ ($C$):

2 $$Estimated\;Mean\;\Delta QTcF\left( C \right) = \theta_{1,Est} + C \times \theta_{2,Est} .$$

Two-sided 90% confidence intervals (CIs) of the estimated ΔQTcF were computed from Eqs. 3 and 4:

3 $$Estimated\;SE = \sqrt {var\left( {\theta_{1,Est} } \right) + C^{2} var\left( {\theta_{2,Est } } \right) + 2C\left( {cov\left( {\theta_{1,Est } \theta_{2,Est } } \right)} \right) }$$

4 $$90\% CI = Estimated\;Mean\;\Delta QTc\left( C \right) \pm t\left( {0.95, DF} \right) \times Estimated\;SE,$$

where $var\left( {\theta_{1,Est} } \right)$ is the variance of the intercept, $var\left( {\theta_{2,Est} } \right)$ is the variance of the slope, and $cov\left( {\theta_{1,Est } \theta_{2,Est} } \right)$ is the covariance of intercept and slope; $t$ is the critical value determined from the t-distribution; DF is the degrees of freedom; SE is the standard error; and CI is the confidence interval.

To exclude a prolongation of QT for lurbinectedin assuming that there is no placebo effect, the upper bound (UB) of the 2-sided 90% CI of the model-predicted mean ΔQTcF had to be lower than 10 ms (threshold set at the ICH E14 Q&A R3 [11]), at the C_max_ geometric mean obtained after administration of the clinically relevant dose of 3.2 mg/m^2^ i.v. over 1-h q3wk.

The C_max_ geometric mean either at the central or at the effect compartment, at which UB 90% CI of the model-predicted mean ΔQTcF would be above 10 ms and 20 ms thresholds, was also estimated according to the formulas provided above.

## Results

From August 2015 to June 2016, a total of 39 evaluable patients were included in the study. At the majority of post-baseline assessments, data were available from at least 35 patients. Most of the 39 patients (*n *= 32; 82.1%) completed all QT assessments; four patients discontinued due to patient refusal, and three patients due to disease progression (one of them died during the study period).

### Patient characteristics

Main patient characteristics are summarized in Table 1. Twenty-two of the 39 included patients (56.4%) were female. Median age was 56 years (range 28–65 years). Blood pressure at study entry was within the limits stated in the inclusion criteria. Performance status score was 0 (*n * = 17; 43.6%) or 1 (*n * = 22; 56.4%). ECG at baseline was normal (*n *= 29; 74.4%) or without significant abnormalities (*n * = 10; 25.6%). Left-ventricular ejection fraction (LVEF) at baseline was within the normal institutional range in all patients.

**Table 1:** Patient characteristics at baseline (*n *= 39)

	*n*	%
Gender		
Female	22	56.4
Male	17	43.6
Age (years)		
Median (range)	56 (28–65)	
28–42 years	5	12.8
43–65 years	34	87.2
Weight (kg), median (range)	76.0 (42.9–115.0)	
Height (cm), median (range)	169.0 (149.0–187.0)	
BSA (m^2^), median (range)	1.9 (1.4–2.3)	
Heart rate (bpm), median (range)	76 (56-103)	
Systolic blood pressure (mmHg), median (range)	123 (93–147)	
Diastolic blood pressure (mmHg), median (range)	74 (56–86)	
Body temperature (ºC), median (range)	36.6 (35.0–37.3)	
ECOG PS		
0	17	43.6
1	22	56.4
ECG		
Normal	29	74.4
Non-significant abnormalities	10	25.6
LVEF, median (range)		
ECHO (*n * = 35)	62.0 (50.0–75.0)	
MUGA (*n * = 4)	65.5 (56.0–67.0)	
Tumor type		
Endometrial carcinoma	9	23.1
Head and neck carcinoma	6	15.4
Neuroendocrine tumors	5	12.8
Small cell lung cancer	5	12.8
Biliary tract carcinoma	4	10.3
Ewing’s family of tumors	3	7.7
Germ cell tumor	3	7.7
BRCA 1/2-associated metastatic breast carcinoma	2	5.1
Carcinoma of unknown primary site	2	5.1

*bpm* beats per minute, *BSA* body surface area, *ECG* electrocardiogram, *ECHO* echocardiography, *ECOG PS* Eastern Cooperative Oncology group performance status, *LVEF* left-ventricular ejection fraction, *MUGA* multiple-gated acquisition scan

The most common tumor types were endometrial carcinoma (*n * = 9; 23.1%); head and neck carcinoma (*n *= 6; 15.4%), and neuroendocrine tumors and small cell lung cancer (*n * = 5 each; 12.8%). Six patients had previously received anthracyclines in the neoadjuvant (*n *= 3) or in the advanced setting (*n * = 4).

With respect to cardiac events at baseline, a 55-year-old female patient diagnosed with endometrial carcinoma had grade 1 diastolic dysfunction, with normal ECG and LVEF values.

### Heart rate correction

**Fig. 1:**
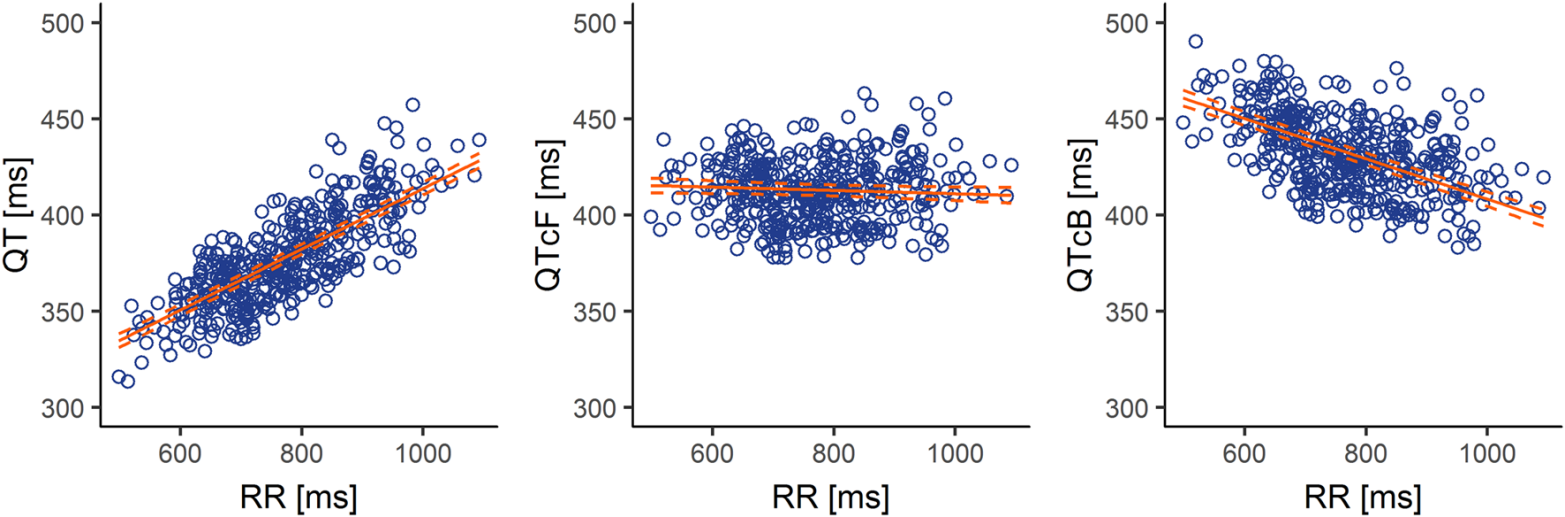
Overlay of mixed-effects model estimates (solid red line) with two-sided 90% CIs (dashed red lines) and observed patient data for QT, Fridericia’s corrected QT (QTcF) and Bazett’s corrected QT (QTcB) *vs.* time-matched RR. Each open circle represents an individual observation at each time point

The performance of Fridericia’s heart rate (HR) correction was compared to that of Bazett’s by graphical analysis of the plots of QT/QTc versus RR intervals (Fig. 1). The Fridericia’s formula corrected for HR reasonably well, with a slight tendency to over-correct resulting QTc values. In contrast, the Bazett’s formula showed a marked over-correction on QT values. QTc versus RR data for both correction methods were also compared by calculating the mean of the R^2^ (95% CIs): − 0.009 (− 0.018 to − 0.001) for QTcF and − 0.105 (− 0.115 to − 0.095) for QTcB. The lower QTcF value with a not significant *p* value different from zero demonstrated that QTcF was less dependent on HR (slope closer to zero) than QTcB, then supporting the selection as per protocol of Fridericia as the primary HR-correction formula.

### Change in QTcF (ΔQTCF)

No patients had pre-dose 2 QTcF value ≥ 20 ms longer than the pre-dose 1, thus ruling out a relevant effect of prophylactic medication on QTcF. As a minimum difference (1.3 ms) was observed between mean pre-dose 1 and mean pre-dose 2 values, the mean of both was used as baseline for the calculation of ΔQTcF.

**Fig. 2:**
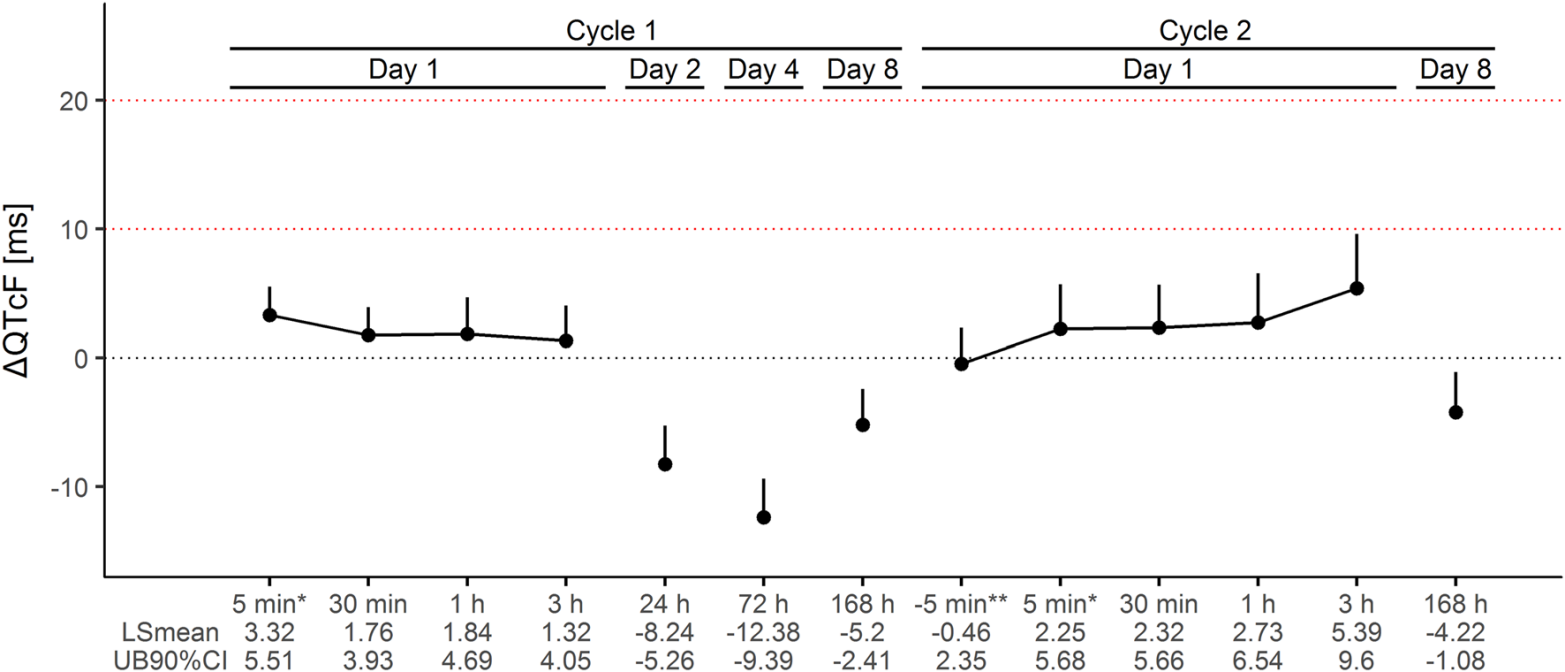
Least squares mean and upper bound (UB) of two-sided 90% confidence intervals (CI) of change in Fridericia’s corrected QT (ΔQTcF) at each time point in cycle 1 and cycle 2, with corresponding values below the horizontal axis. Horizontal dotted red lines represent the 10 and 20 ms threshold criteria. Times at the horizontal axis are after the end of lurbinectedin infusion, saved for those marked with * and ** which are before the end and before the start of lurbinectedin infusion, respectively

LSM of ΔQTcF and two-sided 90% CIs are depicted and summarized in Fig. 2. The maximum LSM ΔQTcF occurred 3 h after the end of cycle 2 infusion (5.39 ms; 90% CI 1.17, 9.60) and, at other time points, LSM ΔQTcF were ≤ 3.3 ms, and UB of the 90% CI were < 6.6 ms. Therefore, the UB 90% CI at all time points were less than the pre-specified cut-off of 20 ms; then, the absence of QTc prolongation by the treatment can be concluded.

### ΔQTcF/lurbinectedin plasma concentration

**Fig. 3:**
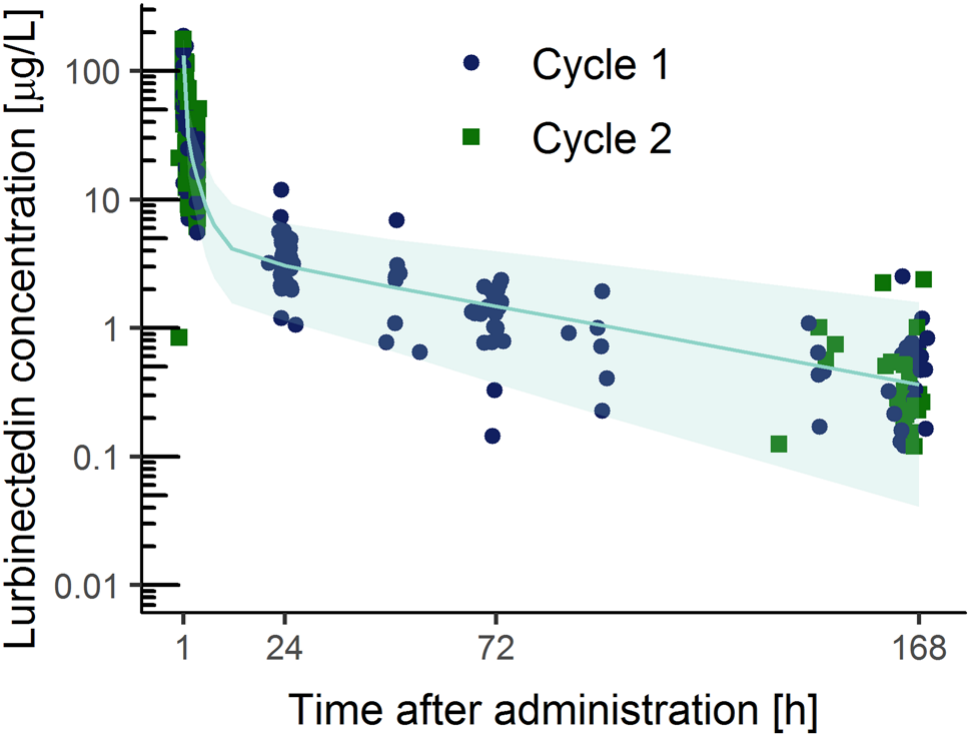
Lurbinectedin plasma concentrations vs. time on cycle 1 and cycle 2. Blue circled and green squared dots represent lurbinectedin plasma concentrations in cycles 1 and 2, respectively. The shaded green area and the green line represent the 90% prediction interval and the median of simulated lurbinectedin plasma concentrations when given at 3.2 mg/m^2^ (from the population PK model) [17]

Mean non-compartmental PK parameters in cycle 1 of the 39 evaluable patients were comparable to that from the population PK model of lurbinectedin containing data from more than 400 patients [4]. Basically, mean (coefficient of variation) clearance was 11.8 L/h (53.3%) and volume of distribution was 347.7 L (51.0%) in the 39 patients, while values of these typical PK parameters were 11.2 L/h and 438.4 L in the population PK analysis, respectively. Mean lurbinectedin plasma concentration was very similar in cycles 1 and 2 (Fig. 3).

**Fig. 4:**
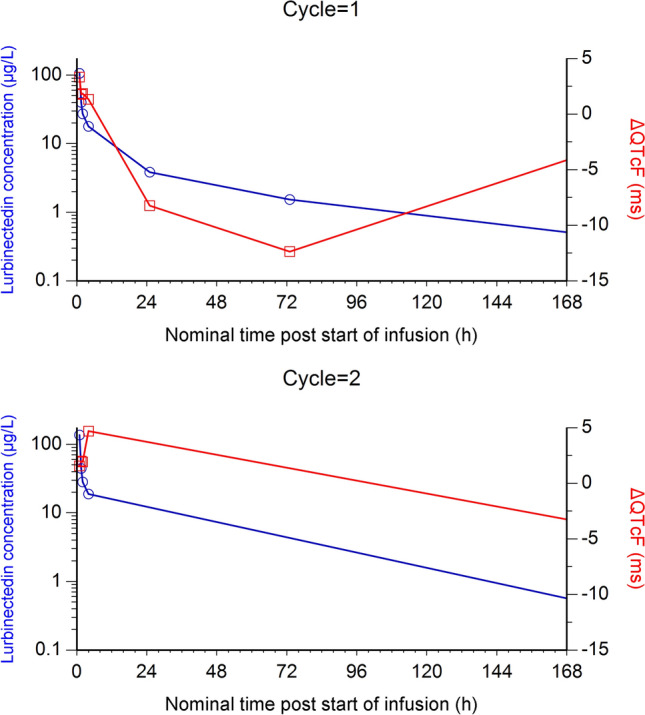
Mean ΔQTcF (red lines, right *y*-axis) and plasma observed lurbinectedin concentration (blue lines, left y-axis), by time point, in cycle 1 (upper panel) and cycle 2 (bottom panel)

Time-matched profiles of mean ΔQTcF and lurbinectedin observed total plasma concentrations are depicted in Fig. 4. The largest mean ΔQTcF was 4.69 ms (at 4 h after the start of infusion on cycle 2), thus not exceeding the 5 ms threshold at any time point (Table 2). Therefore, the first statistical criterion for hysteresis, according to Darpo et al. [10], was unmet. No delay was seen in cycle 1 between U_max_ (time after the administration of a drug when the largest mean ΔQTcF is reached) (0.92 h) and T_max_ (0.92 h), while there was a 3.08 h difference in cycle 2 between U_max_ (0.92 h) and T_max_ (4 h), thus meeting, at least partially, the second criterion for hysteresis. However, in cycle 2, the one-sided one-sample Wilcoxon test for the difference between ΔQTcF at T_max_ (1.54 ms at 0.92 h) and at U_max_ (4.69 ms at 4 h) was not significant at 1% level (*p* value = 0.1749); therefore, the third criterion for hysteresis was not fulfilled. As the presence of a delayed effect of lurbinectedin on the QT interval could not be fully ruled out, direct and indirect effect models were developed to identify and select the best fit to the data.

**Table 2:** Mean (Stdv) ΔQTcF and observed lurbinectedin concentration by time point and Cycle (U_max_, T_max_, and C_max_ in cycle 1 and cycle 2 in italics)

	Cycle 1							Cycle 2				
Time point (h)*	*0.92*	1.5	2	4	25	73	169	*0.92*	1.5	2	4	169
n	38	39	39	39	39	39	34	34	35	34	34	26
Lurbinectedin (µg/L)	*106.02* *(54.52)*	40.01 (24.35)	27.22 (14.05)	17.86 (8.07)	3.84 (1.87)	1.53 (1.13)	0.51 (0.45)	*136.60* *(145.04)*	44.40 25.53	28.22 (16.84)	18.82 (10.94)	0.56 (0.57)
ΔQTcF (ms)	*3.33* *(8.46)*	1.76 (8.25)	1.84 (10.88)	1.33 (10.39)	− 8.24 (11.33)	− 12.38 (11.39)	− 4.05 (8.60)	1.54 (12.36)	1.99 (12.06)	1.92 (13.65)	*4.69* *(15.28)*	− 3.30 (10.47)

*Hours after start of infusion

*ΔQTcF* variation in QTc corrected according to Fridericia’s formula, *C*_*max*_ maximum plasma concentration; *T*_*max*_, time after the administration of a drug when the *C*_*max*_ is reached; *U*_*max*_ time after the administration of a drug when the largest mean ΔQTCF is reached

### Modelling of concentration-ΔQTcF

A model with random effects in slope and intercept and a correlation parameter between these random effects was retained as the final direct model:

5 $$\Delta QTcF_{ij} = \left( {\theta_{1} \times e^{{\eta_{1,i} }} } \right) + \left( {\theta_{2} \times e^{{\eta_{2,i} }} } \right)C_{ij} + {\epsilon_{ij}} .$$

The slope was estimated as 0.072 ms·L/µg, with an intercept of − 1.47 ms (Table 3). Observed and predicted values showed a good agreement. The goodness-of-fit for the residuals did not deviate substantially from normality and no trend was seen in the weighted residuals plots, which showed normal random scatter around zero, without any signals suggesting nonlinearity.

**Fig. 5:**
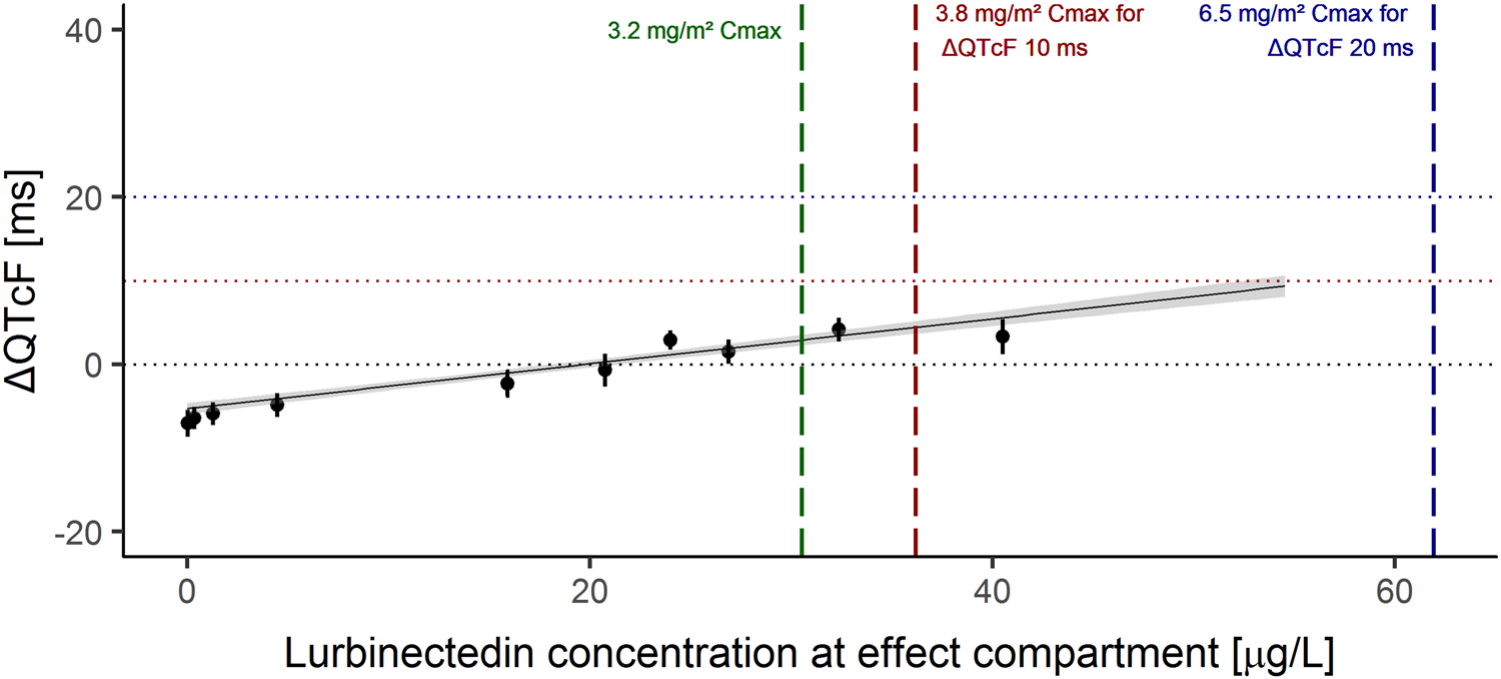
Change from baseline in Fridericia’s corrected QT (ΔQTcF) *vs.* lurbinectedin concentration at effect compartment. The observed ΔQTcF have been grouped into deciles of the predicted concentrations at effect compartment and the average observed ΔQTcF (black dots) have been plotted at the average predicted concentrations in each decile with two-sided 90% CI. Solid black line and grey area denote the model-predicted ΔQTcF with two-sided 90% confidence intervals (CI) as a function of lurbinectedin concentration at the effect compartment. Vertical dashed green, red, and blue lines indicate the maximum plasma concentration (C_max_) geometric mean of lurbinectedin associated with the 3.2 mg/m^2^ dose, C_max_ geometric mean of lurbinectedin associated with the 3.8 mg/m^2^ dose at which upper bound of the two-sided 90% confidence interval of the ΔQTcF is 10 ms (red line), and C_max_ geometric mean of lurbinectedin associated with the 6.5 mg/m^2^ dose at which upper bound of the two-sided 90% confidence interval of the ΔQTcF is 20 ms (blue line)

Nonetheless, an effect compartment model with random effects in slope and intercept showed a major improvement over the corresponding direct effect model. The inclusion of a correlation parameter between the random effects of the slope and the intercept showed an additional improvement. The addition of a cycle effect or a quadratic term to this model was not significant. Therefore, the model selected as final was as follows:

6 $$\Delta QTcF_{ij} = \left( {\theta_{1} \times e^{{\eta_{1,i} }} } \right) + \left( {\theta_{2} \times e^{{\eta_{2,i} }} } \right)Ce_{ij} + {\epsilon_{ij}} ,$$

where *Ce*_*ij*_ is the lurbinectedin concentration at the effect compartment (Table 3). The slope was estimated as 0.289 ms L/µg, the intercept as − 3.95 ms, and the equilibration rate constant (K_eo_) as 0.298 1/h. The linear relationship between ΔQTcF and lurbinectedin concentration at the effect compartment is shown in Fig. 5.

**Table 3:** Parameter estimates for direct model and delayed effect model

MVOF	Final direct model		Final delayed effect model	
	2550		2459	
Parameter	Estimate	RSE (%)	Estimate	RSE (%)
Intercept, ms	− 1.47	8.18	− 3.95	7.22
Slope, ms L/µg	0.072	12.2	0.289	17.6
K_eo_, 1/h	–		0.298	0.292
Residual variability (%)	9.25	3.83	8.36	3.92
Variability in intercept (%)	165	13.1	105	13.3
Variability in slope (%)	14.0	73.9	29.8	40.3
Correlation intercept-slope (%)	− 49.5	29.4	− 63.5	25.8

*K*_*eo*_, equilibration rate constant; *MVOF* minimum value of the objective function; *RSE* relative standard error

**Fig. 6:**
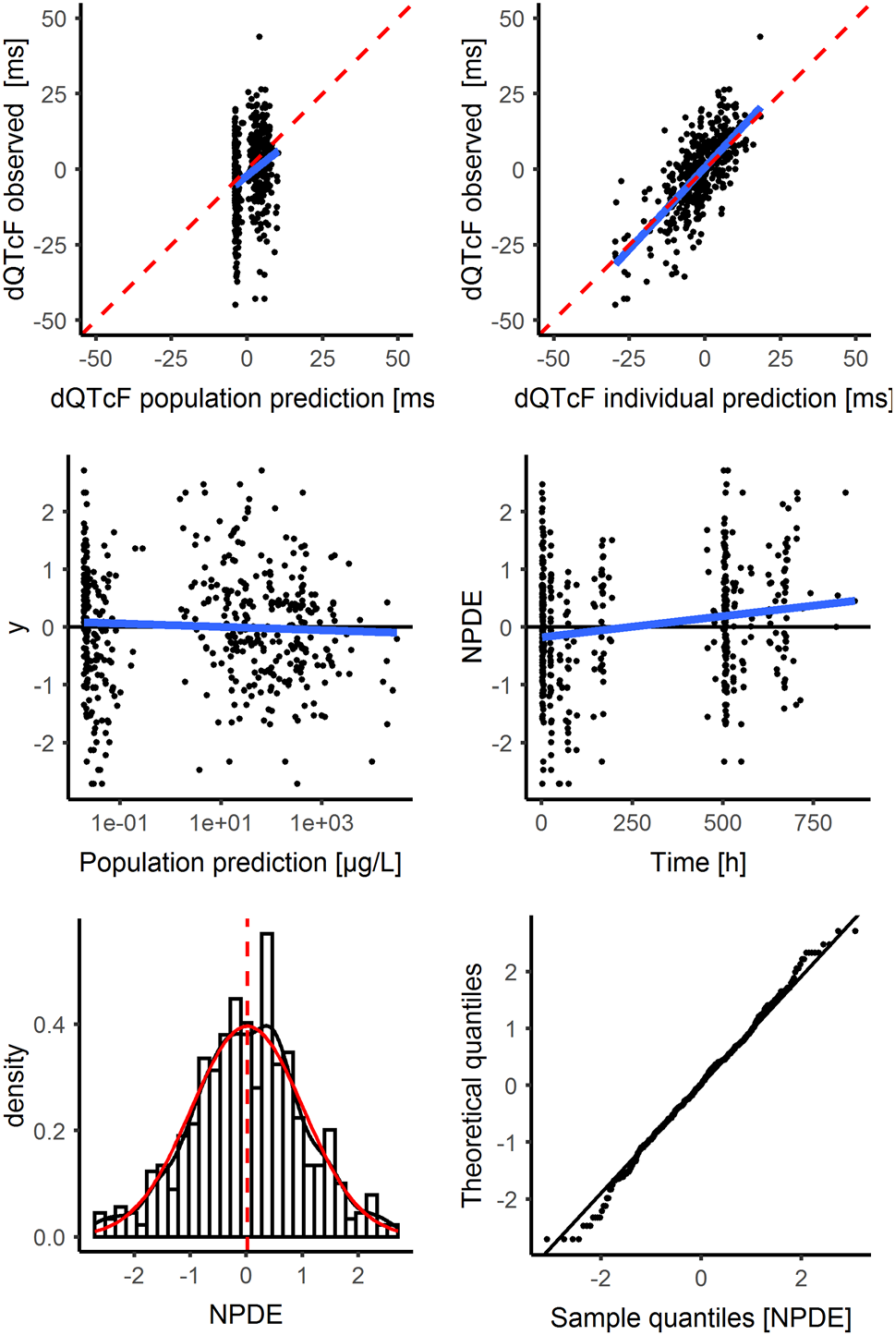
Diagnostic plots of the linear mixed effect model for the delayed concentration–ΔQTcF relationship. Upper panels: observed vs. population (left panel) and individual (right panel) predicted plots. *Red dashed line* line of unity, *blue line* loess smoother. Middle panels: normalized prediction distribution errors (NPDE) vs population predicted (left panel) and time (right panel). *Black line* line of unity, *blue line* loess smoother, *dashed lines* upper and lower limits. Lower panels: histogram (left panel) and QQ plots (right panel) for normalized prediction distribution errors (NPDE). *Red line* probability density function from the data, *dashed red line* mean, *black line* probability density function from a Gaussian distribution based on the data

The appropriateness of the indirect model was also explored graphically, without any signals suggesting nonlinearity (Fig. 6).

The predicted ΔQTcF at C_max_ of lurbinectedin at the effect compartment (30.53 µg/L) at the recommended dose was 4.87 ms (90% CI 1.94–7.81). With the direct effect model (C_max_ of 116.62 µg/L), ΔQTcF was 6.93 ms (90% CI 4.96–8.90). Therefore, regardless of the direct or indirect effect of lurbinectedin concentrations, ΔQTcF at the recommended dose is not expected to exceed 10 ms.

Moreover, C_max_ of lurbinectedin at the effect compartment associated with the thresholds of 10 and 20 ms UB 90% CI of ΔQTcF were estimated for the delayed effect model as 36.18 and 61.94 µg/L, respectively. Based on C_max_ at the recommended dose (30.53 µg/L), 36.18 and 61.94 µg/L would correspond to lurbinectedin dose of 3.8 mg/m^2^ and 6.5 mg/m^2^, respectively, which represent a 1.21- and 2.03-fold increase. With the direct effect model, C_max_ associated with those thresholds were estimated as 129.05 and 241.25 µg/L, respectively, corresponding to doses of 3.6 and 6.6 mg/m^2^.

### Change in other ECG parameters

As with the QTc interval, mean (± Std) values of other ECG parameters remained constant along the study except for HR, which showed a transient increase with the highest change from baseline at 3 h after the end of infusion: 16.7 (± 11.0) bpm in Cycle 1 and 17.5 (± 11.7) bpm in cycle 2. Mean change from baseline of PR interval and QRS duration showed limited fluctuation across all time points and cycles, with minimal variations between the lowest and largest mean values of 7.8 ms and 2.5 ms, respectively.

### Safety

No relevant cardiac safety findings were observed within the QT evaluation study period, except for one case of grade 3 hypokalemia (potassium of 2.5 mmol/L) occurred in a patient with grade 4 respiratory failure and aspiration not related to the study treatment, which was resolved after i.v. electrolyte replacement.

## Discussion

This study was designed to evaluate the potential effect of lurbinectedin at the recommended dose (3.2 mg/m^2^ given as a 1-h i.v. infusion q3wk) on the QT interval duration following, to the extent feasible, current regulatory standards described in the ICH E14 guideline [12]. Possible immediate or delayed effect for delaying cardiac ventricular repolarization was assessed through centralized, blinded, third-party evaluation of changes in QTc during treatment at thirteen post-baseline time points. Thirty-nine evaluable patients were included in this study and they provided a total of 1707 ECGs, with most of the scheduled ECG assessments available in at least 35 patients.

The method for heart rate correction (Fridericia) selected for the primary endpoint in this study was found to be more accurate than the Bazett’s correction method (R^2^ for QTcF and QTcB vs. RR of − 0.009 and − 0.105, respectively), which has been already reported to over-correct the QT interval with increasing HR, resulting in false positives for QTc prolongation [13], and is, no longer, warranted unless used for specific reasons [12]. Moreover, the Fridericia’s correction tends to be even more reliable and accurate than the Bazett’s correction when compensating for changes in heart rate induced by a drug or by clinical conditions. This is especially relevant when the heart rate increases during therapy as compared with baseline [7].

The primary endpoint analysis in this study consisted of a “by time point” analysis of change in QTcF (ΔQTcF). UBs of the two-sided 90% CIs of ΔQTcF at any time point did not exceed the pre-defined 20 ms non-inferiority margin, i.e., the threshold typically employed for oncology agents [7]. The largest UB of 90% CI corresponding to 3 h after end of infusion at Cycle 2 was 9.6 ms, thus lower than the more conservative 10 ms threshold established in the ICH E14 guideline for thorough QT studies in healthy volunteers [12]. Of note, in a human mass balance study (EudraCT No. 2016-000800-27), the exposure of all lurbinectedin metabolites when compared to that of the parent compound was considerably lower (10% or less) and their half-lives and tmax were similar or even shorter, thus ruling out their contribution to the apparent 3-h delay of the largest UB 90% CI of ΔQTcF. No single value was above the reference limit values of concern (namely, QTcF > 480 ms and ΔQTcF > 60 ms) in any of the 39 evaluable patients. Therefore, a large effect of lurbinectedin on QT interval is confirmed as very unlikely.

In a secondary endpoint analysis, the relationship between ΔQTcF and time-matched exposure to lurbinectedin was evaluated; this analysis aimed to improve the evaluation by means of gathering in the same model all ΔQTcF values collected across the wide range of lurbinectedin plasma concentrations. The fit between lurbinectedin concentrations and ΔQTcF was improved when an effect compartment was added, and a slightly positive slope (0.289 ms·L/µg) was found between the concentration at the effect compartment and ΔQTcF (of note, lowest lurbinectedin concentrations from the terminal phase were related to negative mean ΔQTcF values, thus leading to a likely overestimation of the resulting positive value). The UB 90% CI of ΔQTcF was estimated to be 7.81 ms at C_max_ at the effect compartment, following the recommended dose. As proposed by Darpo et al. [10], and later acknowledged at the ICH E14 Q&A R3 [12], a clinically relevant QT effect can be excluded when the UB of the two-sided 90% CI for the QTc effect is below 10 ms at plasma levels of the compound that can be observed at the highest clinically relevant exposure, to conclude that an expanded ECG safety evaluation during later stages of drug development is not needed. According to this criterion, lurbinectedin is not associated with an effect of concern on cardiac repolarization. Furthermore, based on the relationship established, a dose of 6.7 mg/m^2^ i.v. over 1-h q3wk is the expected maximal dose that will not exceed the threshold of 20 ms, established for oncology drugs.

For compounds that increase or decrease heart rate more than 10 bpm, subject-specific QT–RR correction methods to account for heart rate effects are encouraged [14]. The superiority of these methods over the Fridericia formula rely on the collection of either drug-free QT data in all subjects over a wide range of heart rates, which implies the inclusion of a baseline (drug-free) day, or 5-min history of preceding RR intervals for each QT interval measurement by means of Holter ECGs. To avoid longer inpatient confinement or troublesome procedures, the present study did not incorporate any of these assessments, which may suppose a limitation on the correction of heart rate effects.

In this study, a modest increase of mean heart rate (maximum ΔHR of 16.7 bpm in Cycle 1 and 17.5 bpm in Cycle 2) affecting an ample number of patients (44% in Cycle 1 and 49% in Cycle 2, with ΔHR > 25%) was typically detected at 4 h after the start of the 1-h infusion, thus not related to C_max_, and baseline values were recovered in the next assessments. This finding had been already reported in previous clinical trials. Moreover, a similar pattern is also described for trabectedin, the first-in-class RNA polymerase II inhibitor; in a dedicated QT evaluation study with this compound given as a 3-h i.v. infusion [15], heart rate increased slightly, with largest increases from baseline at 4 h after the start of infusion, and declining to values similar to baseline at 24 h. No clinical consequence was observed with either drug. Trabectedin, an approved therapy for the treatment of soft-tissue sarcoma and ovarian cancer, is known to have a low cardiac risk profile [16]. This transient increase in heart rate observed with both compounds, whether direct (e.g., sympathomimetic effect) or indirect (e.g., vasodilatation, autonomic tone or anxiety), is of unknown significance.

No significant effects either on atrioventricular conduction or on depolarization, as measured by mean changes in PR and QRS intervals, were observed. No adverse events suggestive of proarrythmic potential were reported.

In conclusion, ECG parameters and concentration–ΔQTc modelling in this prospective study indicate that lurbinectedin is not associated with a clinically relevant effect on cardiac repolarization. Hence, lurbinectedin treatment is not likely to be associated with signals of clinical concern in the development of *torsades de pointes*/sudden death.
